# Spatial activity mapping of ß-mannanase on soybean seeds

**DOI:** 10.1038/s41598-024-51494-w

**Published:** 2024-01-10

**Authors:** Markus Rueckel, Sven Janson, Arne Solbak, Anna Fickler

**Affiliations:** 1 BASF SE, Carl-Bosch-Straße 38, 67056 Ludwigshafen, Germany; 2 BASF Enzymes LLC, 3550 John Hopkins Court, San Diego, CA 92121 USA

**Keywords:** Cellular imaging, Fluorescence imaging, Dietary carbohydrates

## Abstract

For farm animals the supplementation of exogenous enzymes, like ß-mannanase, to soybean-based diets is beneficial to improve feed digestibility. In order to unravel the effect of ß-mannanase on soybean meal’s cell structure, a novel imaging concept was developed which allows visualizing the spatial activity pattern of ß-mannanase with high sensitivity by fluorescence microscopy before any visible degradation of the cellular structure occurs. It is based on fluorescence labeling of newly formed reducing ends of ß-mannanase-hydrolyzed polysaccharides after the native reducing ends of all polysaccharides present were chemically reduced. It was revealed that ß-mannanase is not only active at the cell wall but also at previously unknown sites, like the middle lamella and, most prominently, at an intracellular matrix enclosing the protein storage vacuoles. Based on these findings it can be hypothesized that the evaluated ß-mannanase can degrade the enclosing matrix of encapsulated proteins and the cell wall structure and thereby improves efficiency of feed utilization.

## Introduction

Intensive production of farm animals for generation of meat, milk and eggs can be enhanced by improved feed utilization, leading to less consumption of natural resources and less excretion of undigested nutrients and thus reduces the adverse impact on the environment. Monogastric farm animals, like poultry and swine, typically lack endogenous enzymes to break down non-starch polysaccharides, such as cellulose and hemicellulose^1^. The supplementation of exogenous enzymes, predominantly glycoside hydrolases, to animal diets can make a significant contribution to improving digestibility and feed utilization by maximizing the bioavailability and by alleviating the anti-nutritional components of animal feed^2,3^. Among these glycoside hydrolases, ß-mannanase is one of the most relevant feed enzymes currently in use, in particular for mannan-rich diets, consisting typically of a mixture of corn and soybean meal (SBM)^4^. Galactomannan, an important fraction of the hemicellulose family, is the most abundant mannan species in soybean meal and consists of a polymannose backbone with galactose side groups^5,6^.

The supplementation of ß-mannanase to a soybean-rich feed could lead to a combination of several positive effects^7^: it can reduce the digesta viscosity in the gut of monogastric animals by hydrolyzing long-chain ß-mannans (~ 10^6^ Da), highly branched galactomannans with strong water binding capacity^8,9^. In addition, these hydrolyzed ß-mannans could function as an additional energy source. Furthermore, the release of short ß-1,4-manno-oligosaccharides could suppress the proliferation of harmful microorganisms in the gut and, at the same time, could procure a beneficial prebiotic effect. Moreover, the degradation of ß-mannans could reduce the stimulation of the innate immune response, leading to higher animal performance^10^, which was demonstrated for ß-mannanase-supplemented corn-SBM based diets^11^. The last effect, termed anti-caging effect, relates to the boosted release of trapped or bound nutrients by the degradation of mannan-associated hemicelluloses in the cell wall^12,13^. Sundu et al. reported that ß-mannanase supplementation to mannan-rich corn-SBM-copra meal-based diets significantly improved protein and lipid digestibility in poultry^14^. Several studies evaluated the potential modes of action of ß-mannanase and found inconsistent effects or could only speculate about the impact of ß-mannanase. Furthermore, they did not focus directly on the effect of ß-mannanase on SBM, which is one of the commercially most relevant contributor to dietary ß-mannans in monogastric feed. Therefore, the objective of our study is to elucidate the degradation sites of ß-mannanase on SBM by using a novel sensitive method.

The mechanism of how glycoside hydrolases, to which ß-mannanase belongs to, act on such diets was frequently studied by visualizing structural changes of the degradation process using fluorescence microscopy. The sites, where the hydrolases were active, were monitored after enzyme treatment either by a reduced staining efficiency using fluorescently-labeled antibodies against specific polysaccharides^12,15,16^ or by a release of fluorescently-stained (using e.g. histochemical dyes) or autofluorescent cell structures^15,17,18^. However, both strategies cannot reveal enzyme activity on structures which are not fluorescently stained or are not intrinsically fluorescent and they lack sensitivity in monitoring hydrolysis on a molecular scale, which does not necessarily lead to structural changes, visible by fluorescence microscopy. In case of the immunofluorescence approach several further detrimental effects need to be considered: epitopes of polysaccharides can be masked, antibody cross-reactivity with other polysaccharides is cumbersome to exclude and the large size of IgM-type antibodies, which is the case for most antibodies that target polysaccharides, could render them less accessible^19,20^. Another approach to unravel the interaction of hydrolases with their substrates is by fluorescence labeling of the hydrolases themselves using small fluorescent dyes^21–24^. In these cases, the adsorption sites of the fluorescently labeled hydrolases can be identified but it cannot be assessed directly whether the hydrolase is active and degrades ß-mannans at the adsorbed site.

We will present here a novel and sensitive fluorescence-based concept to elucidate at which sites on the substrate glycoside hydrolases are active and how fast they hydrolyze there. The concept is based on selective fluorescence labeling of newly formed chain ends of polysaccharides cleaved by the hydrolase and thus allows monitoring changes on a molecular scale which occurs before significant structural degradation becomes visible. We apply this novel concept to visualize the interaction of ß-mannanase with soybean seeds and meal and, thereby, unravel at which cellular structures ß-mannanase is active.

## Methods

### Reduction step & Fluorescence labeling for soybean seeds

For studies on soybean seeds (purchased from Rapunzel (https://www.rapunzel.de/), cultivar: 'Amandine'), the seeds were immersed in water overnight, the soybean hull was removed and were then broken apart into their two halves. Afterwards the soybean halves were reduced by 0.1% sodium borohydride (NaBH_4_, reducing agent, Sigma-Aldrich, 452882) in water (pH 7) for 1 h and afterwards incubated in hard water (14° dH, pH 6.8) with 10 ppm CF® 633 Dye Aminooxy (Biotium, #92053) and 1000 ppm aniline (Sigma-Aldrich, 242284) for about 30 min^25^. Subsequently, 1000 ppm (w/w) of an endo-1,4-ß-D-mannanase concentrate (Natupulse® TS, BASF SE, VDD19287.1, GH5 family; CAZy http://www.cazy.org), expressed in *Thermothelomyces thermophilus*, was added to the dye solution and kept for 20 h at room temperature^26^. All concentrations are related to the weight of the aqueous solution. Control experiments were conducted without ß-mannanase but otherwise under the same conditions. Finally, the soybean halves were washed several times with water in order to remove unbound dye.

### Reduction step and Fluorescence labeling for SBM

500 mg of defatted soybean meal (44% crude protein content), was suspended in 15 ml of an aqueous solution with 0.4% NaBH_4_ at pH 7 for 24 h. Afterwards the reduced soybean meal was washed five times in Milli-Q water and two times with hard water (14° dH, pH 6.8). The experiments with 5000 ppm Natupulse® TS (corresponds to about ~ 450 TMU/g of SBM) were conducted at 40 °C for 2 h, the other experiments with 1000 ppm Natupulse® TS (~ 90 TMU/g of SBM) and with 80 ppm Natupulse® TS (~ 7 TMU/g of SBM) were done at room temperature. All concentrations are related to the amount of soybean meal. As indicated in the results section, either in-situ or ex-situ studies were conducted. For in-situ studies, the imaging process was directly started after ß-mannanase was added to the soybean meal suspension. Ex-situ studies were done on a lab mixer (Eppendorf, Thermo Mixer C) at the indicated temperature. Control experiments without ß-mannanase were done under the same conditions.

### Staining of SBM by Calcofluor white and Nile red

SBM was suspended in an aqueous solution either of 1000 ppm (ready to use solution) Calcofluor White (Sigma-Aldrich, 18909) or of 100 ppm Nile Red (Sigma Aldrich, N3013, from a 1000 ppm stock solution in DMSO). Concentrations are again related to the amount of soybean meal.

### Fluorescence labeling of ß-mannanase and adsorption on soybean seed and meal

3 mg of ATTO655-NHS ester (Sigma-Aldrich, 76245) together with 1 ml of Natupulse® TS reacted for 3 h in 1 ml DMSO. After dialysis in water 4 ml of labeled Natupulse® TS was obtained (concentration was lower by a factor of 4). After 2 h incubation of 100 ppm fluorescently labeled Natupulse® TS with soybean meal xyz-stacks were taken at different spots.

In order to compare whether the chemical reduction has an impact on the adsorption behavior of ß-mannanase, a soybean section was reduced by 0.1% NaBH_4_ in water (pH 7) for 1 h, then washed several times in Milli-Q water and finally incubated with 100 ppm of fluorescently-labelled ß-mannanase for 2 h. For the non-reduced section the incubation conditions were the same.

### Fluorescence imaging parameters

For imaging, using an inverted confocal fluorescence microscope (Leica, SP8), the prepared soybean meal suspension or the soybean half was put into a Lab-Tek™ chamber (Nunc™ Lab-Tek™ II Chambered Coverglass, No 1.5 borosilicate glass, sterile, 155,409, Thermo Fisher Scientific Inc.). CF® 633 Dye was excited at 633 nm and the emission was collected between 640 and 800 nm using a water-immersion objective lens (HC FLUOTAR L 25x/0.95 W VISIR, Leica). For in-situ studies of the ß-mannanase activity xyzt-scans were recorded with z-steps of 0.5 µm. For ex-situ studies xyz-stacks were taken and processed as indicated in the figure captions.

Soybean meal, stained by Calcofluor white, was excited at 405 nm and the emission was collected between 410 and 465 nm. For Nile red stained soybean meal emission was recorded between 570 and 750 nm for an excitation wavelength at 561 nm. For both studies a high-resolution objective lens (HCX PL APO 63x/1.20 W, Leica) was used.

The fluorescently labeled ß-mannanase was excited at 633 nm and the emission was collected between 640 and 800 nm.

All methods were carried out in accordance with relevant guidelines and regulations.

## Results

### Validation of novel visualization concept

**Figure 1:**
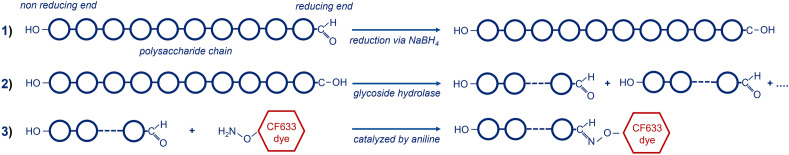
The visualization concept of the activity of glycoside hydrolases is based on three steps: (1) chemical reduction of reducing terminal ends of all polysaccharide chains present, (2) glycoside hydrolases hydrolyze partially specific polysaccharides and new reducing ends are formed which become fluorescently labeled by aminooxy-bearing dye, CF® 633 Dye Aminooxy, catalyzed by aniline (step 3).

The concept to visualize the activity of glycoside hydrolases on polysaccharides, distributed in the cellular tissue, is based on selective labeling of newly formed reducing ends by the hydrolase-triggered cleavage of glycosidic bonds. At the reducing ends of polysaccharides aldehyde or keto groups of hemiacetals in their open form can be covalently attached to a fluorescent dye via an aminooxy-functionality^27^. The formation of the resulting oxime linkage can be catalyzed by addition of aniline^28^. However, every polysaccharide chain offers already in its native, uncleaved form a reducing terminal end which would become directly labeled by the aminooxy-bearing dye. Therefore, in order to spatially map the cleavage activity of glycoside hydrolases, the native reducing ends of all polysaccharide chains are reduced chemically first to hydroxyl groups which cannot react with the aminooxy-bearing dye. The concept is therefore based on three steps: (1) chemical reduction of the reducing ends of all polysaccharides present, (2) activity of glycoside hydrolases lead to the formation of new reducing ends which react in step (3) with the aminooxy-bearing dye (Fig. 1). Therefore, all the sites at the substrate, where glycoside hydrolases are active, are potentially fluorescently labeled.

**Figure 2:**
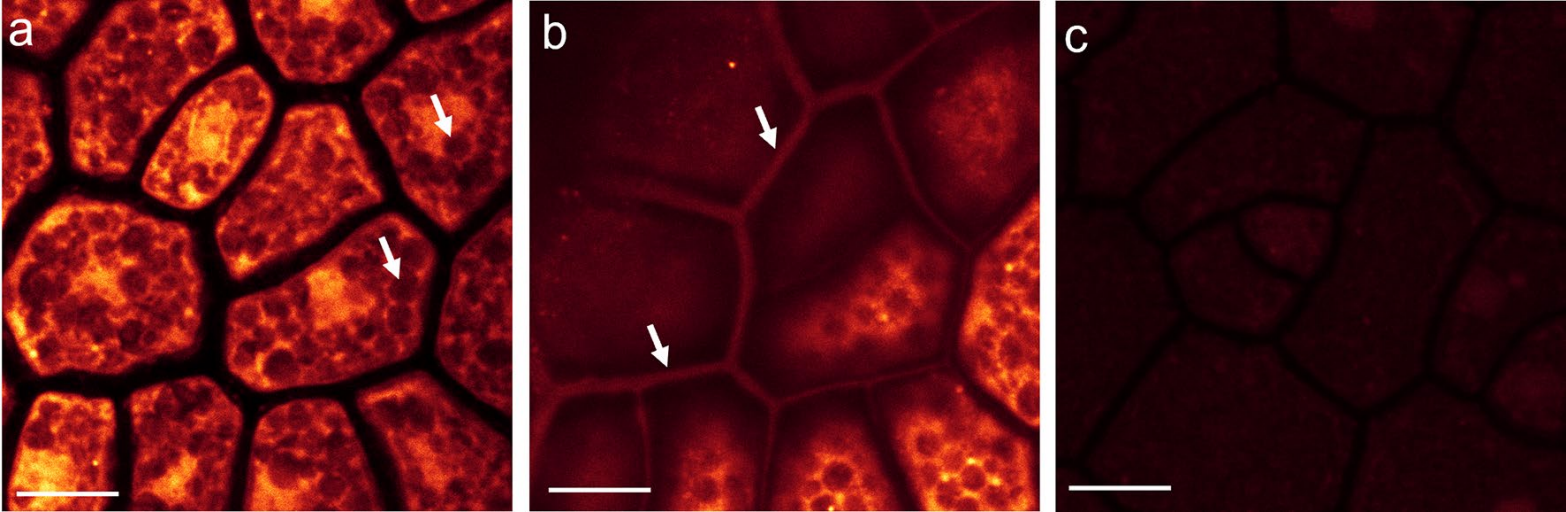
Single sections from a xyz-stack of the fluorescence distribution in endosperm cells of a soybean seed after 20 h incubation with 1000 ppm ß-mannanase (Natupulse® TS) and 10 ppm CF® 633 Dye Aminooxy. Unbound dye was washed off in several steps. (**a**) Deep inside the tissue fluorescence could only be detected intracellularly but neither at the cell wall nor within the protein storage vacuoles (dark compartments, marked exemplarily by white arrows). (**b**) Close to the surface of the soybean half cell walls were weakly labeled (marked exemplarily by white arrows). Since the focal plane was inclined to the surface of the soybean half, the focal plane was deeper within the tissue on the bottom right of the image. (**c**) The control experiment without ß-mannanase, executed and imaged under the same conditions, showed only a very weak fluorescence signal due to autofluorescence. Scale bar is 10 µm and the axial distance between (**a**) and (**b**) is 6 µm.

To validate this novel concept, we visualized the activity of ß-mannanase on matured soybean seeds. When disunited and chemically reduced soybean halves were treated by ß-mannanase in presence of the aminooxy-bearing dye for 20 h, the fluorescence labeling was strong within the cell and weak at the cell wall (Fig. 2a,b). Only at the surface of the soybean half, cell walls became fluorescent, whereas deeper inside the tissue, cell walls were not fluorescent. The dye can penetrate a few cell layers deep into the soybean tissue (see SI, video 1) and therefore it suggests that ß-mannanase was not active at the cell walls deeper inside the tissue. Intracellularly, a cytoplasmic matrix, enclosing the protein storage vacuoles (PSV), became strongly fluorescent. No visible degradation and release of cellular structures could be observed. Control experiments without ß-mannanase but with the same preparation and imaging conditions did not show any fluorescence labeling of such structures and only a weak background fluorescence signal was detected (Fig. 2c). Additionally, this novel visualization concept of mapping glycoside hydrolase activity was validated for a cellulase on cotton tissue (see SI, video 2).

### Interaction of ß-mannanase with SBM

**Figure 3:**
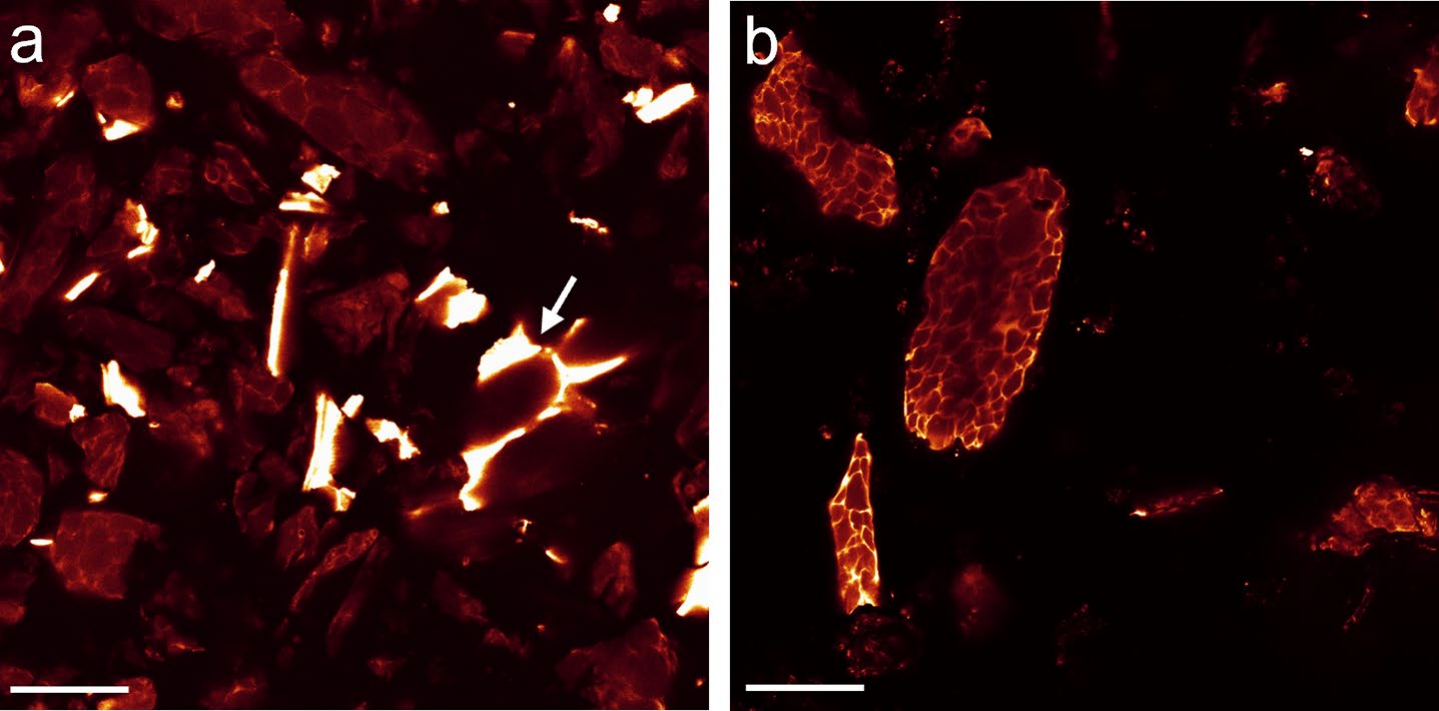
SBM after staining with (**a**) Calcofluor white and (**b**) Nile red. Calcofluor white predominantly stained the cell walls (oversaturated intensity, marked by white arrow) but also weakly the intracellular structure around the PSVs. Nile red stained only the intracellular matrix. Scale bar is 40 µm.

As a next step, the visualization concept was applied onto SBM, the most relevant substrate of dietary ß-mannanase^29,30^. Because of preprocessing steps like oil extraction and milling, most of the cellular structures of SBM were partially disintegrated and deformed, with only fragments of soybean tissue remaining (Fig. 3). Fragments of cell walls, cell wall structures without cytoplasm, soybean hull and cytoplasmic structures without cell walls could be identified in SBM by their morphology and staining characteristics. For example, Nile red, a lipophilic dye to stain lipid droplets^31^, showed a strong affinity towards the intracellular matrix enclosing the PSVs.

**Figure 4:**
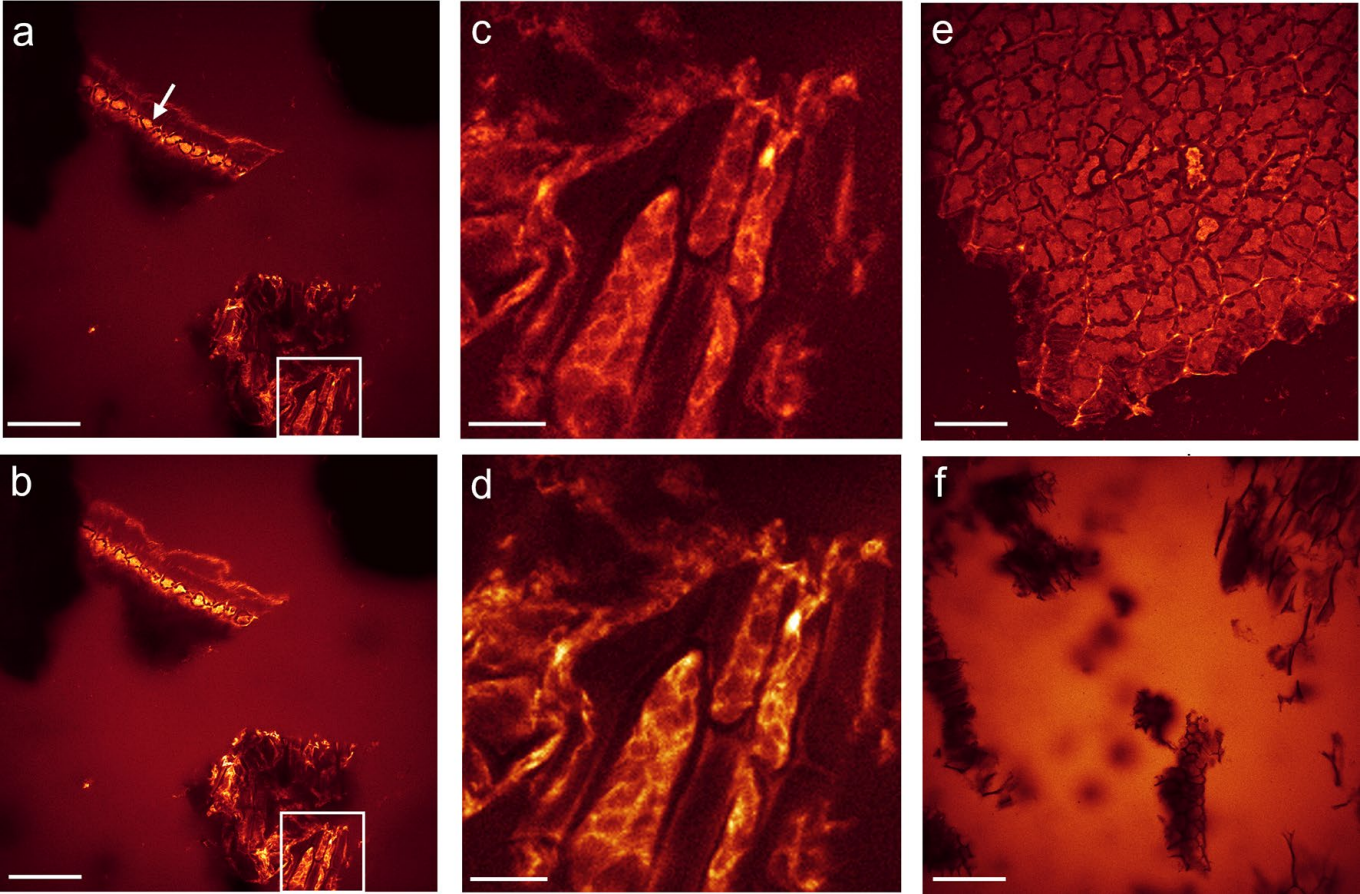
SBM in an aqueous solution of CF® 633 Dye Aminooxy after chemical reduction by NaBH_4_. (**a**) Directly after addition of 1000 ppm ß-mannanase (Natupulse® TS) cellular structures like cell walls and intracellular domains were stained strongly (see lower right corner). Soybean hulls were strongly stained, too (marked by white arrow). Because of the high inclination angle of the soybean hull fragment in relation to the focal plane only a thin strip of the hull structure was in focus. (**b**) The same location as in (**a**) but 1 h later. White-outlined region in (**a**) and (**b**) were magnified in (**c**) and (**d**), respectively, to highlight the intracellular structures. Please notice the increase in fluorescence intensity of the stained structures of about 50% after 1 h of ß-mannanase activity. (**e**) Magnified maximum projection of a xyz-stack of the marked hull structure in (**a**). (**f**) Without ß-mannanase CF® 633 Dye Aminooxy remained in solution and did not stain any cellular structures after 5 h. Scale bar is 100 µm for (**a**), (**b**) and (**f**), 50 µm for (**e**) and 20 µm for (**c**) and (**d**).

Monitoring the activity of a high concentration of ß-mannanase (here 1000 ppm of Natupulse® TS, which is much higher than the recommended dose of about 10 ppm) on SBM revealed similar ß-mannanase activity patterns as found in matured soybean seeds (Fig. 4). Again, cellular structures of SBM appeared non-fluorescent after chemical reduction of the polysaccharides’ reducing ends without ß-mannanase application. Only a background fluorescence of dissolved CF® 633 Dye Aminooxy in the aqueous medium was observable (Fig. 4f), leading to a negative imaging contrast of the SBM fragments. Strong activity was identified again intracellularly around the PSVs and on the soybean hull when ß-mannanase was supplemented (Fig. 4a–e).

**Figure 5:**
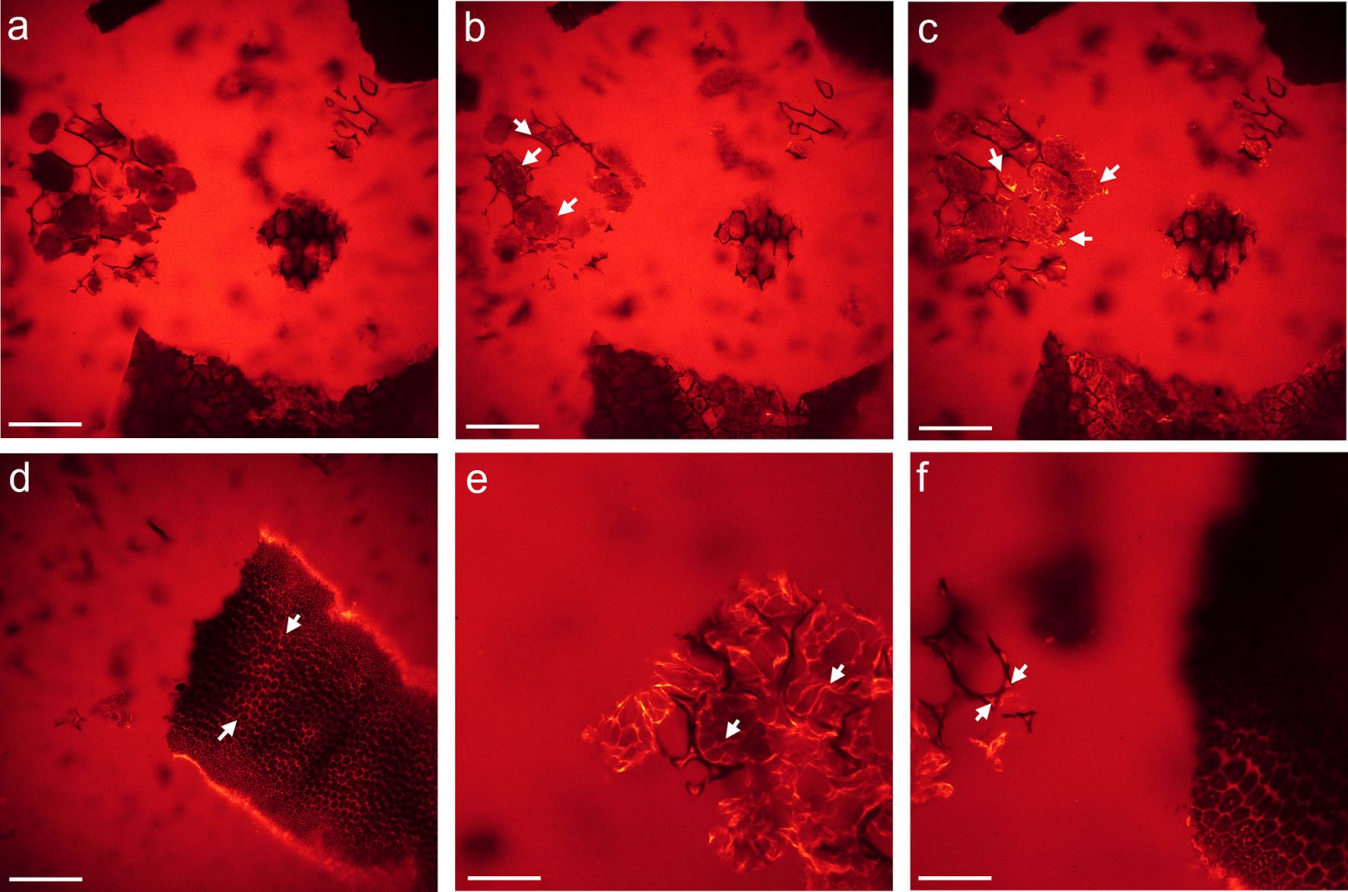
In-situ study of 80 ppm ß-mannanase (Natupulse® TS) on SBM after chemical reduction by NaBH_4_. (**a**) Before addition of ß-mannanase no cellular structures were labeled by CF® 633 Dye Aminooxy. (**b**) After 60 min of addition of ß-mannanase first intracellular structures were stained. (**c**) After 130 min the intracellular matrix enclosing the PSVs became fluorescent clearly. After 24 h ß-mannanase was active at (**d**) the cell wall structure of the hull, (**e**) intracellularly and (**f**) at the middle lamellar structure. All structures mentioned were marked exemplarily by white arrows Scale bar for (**a**)–(**d**) is 100 µm and (**e**)–(**f**) is 40 µm. See also video 4&5 of SI.

**Figure 6:**
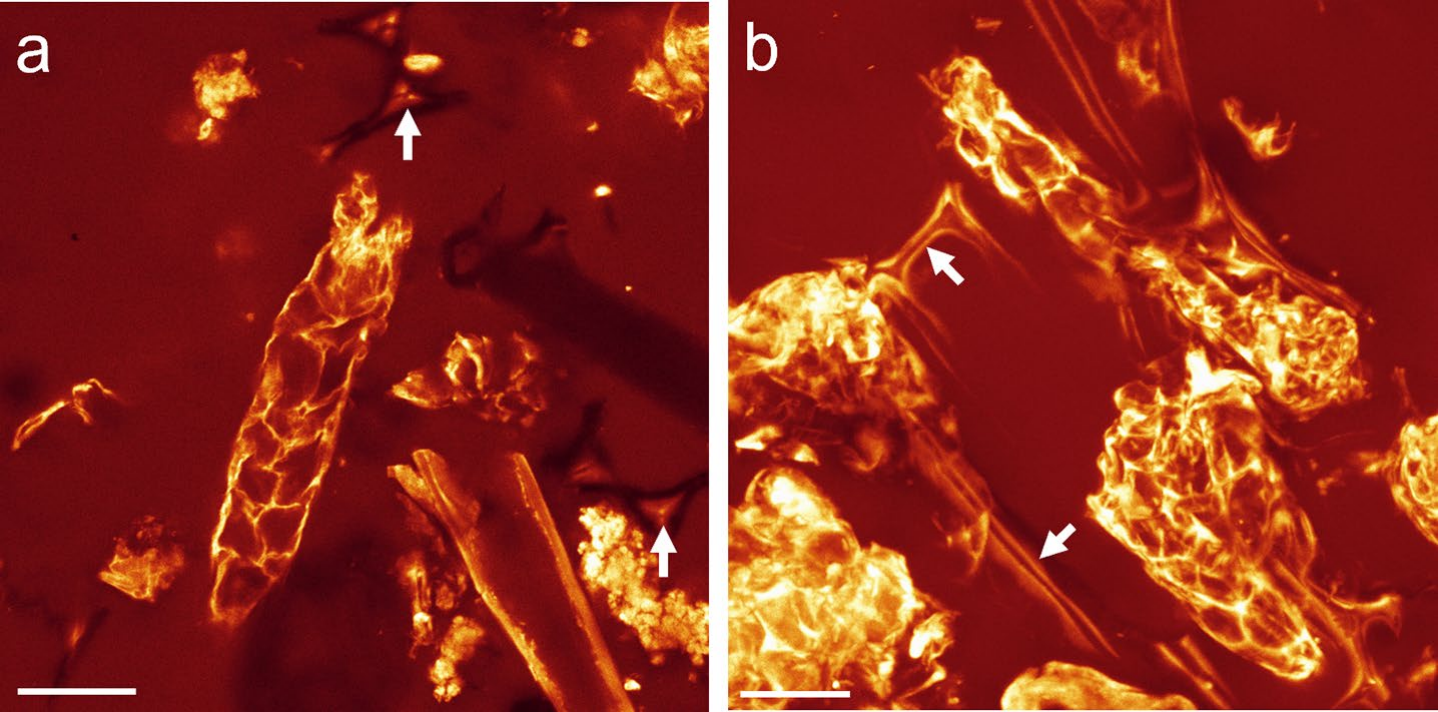
SBM in an aqueous solution of CF® 633 Dye Aminooxy after chemical reduction by NaBH_4_ and 2 h treatment with 5000 ppm ß-mannanase (Natupulse® TS) at 40°C. Apart from the fluorescent intracellular structures, either (**a**) the middle lamella or (**b**) the cell walls became fluorescent due to the action of ß-mannanase (marked exemplarily by white arrows), presumably representing different cell types of the soybean seed. Scale bar is 25 µm for (**a**) and 15 µm for (**b**).

By visualizing in-situ the activity of a lower dose of 80 ppm ß-mannanase, the kinetics of the hydrolysis process can be monitored. It took about 1 h of incubation in order to identify the first activity sites of ß-mannanase under our chosen conditions at room temperature and at a neutral pH, at which the fluorescence signal became enhanced intracellularly again (Fig. 5). However, the fluorescence staining of cell wall structures was significantly lower than of the intracellular matrix. Strikingly, an increased fluorescence signal at the middle lamella, where two cells adjoin, appeared after about 24 h treatment (Fig. 5f). In contrast to the previous experiment, no fluorescence enhancement was detected directly at the cell walls. Under slightly different conditions, with higher ß-mannanase concentration and treatment at 40 °C, both the cell walls and the middle lamella became strongly fluorescent, however, not at the same cells (Fig. 6).

### Control experiments

**Figure 7:**
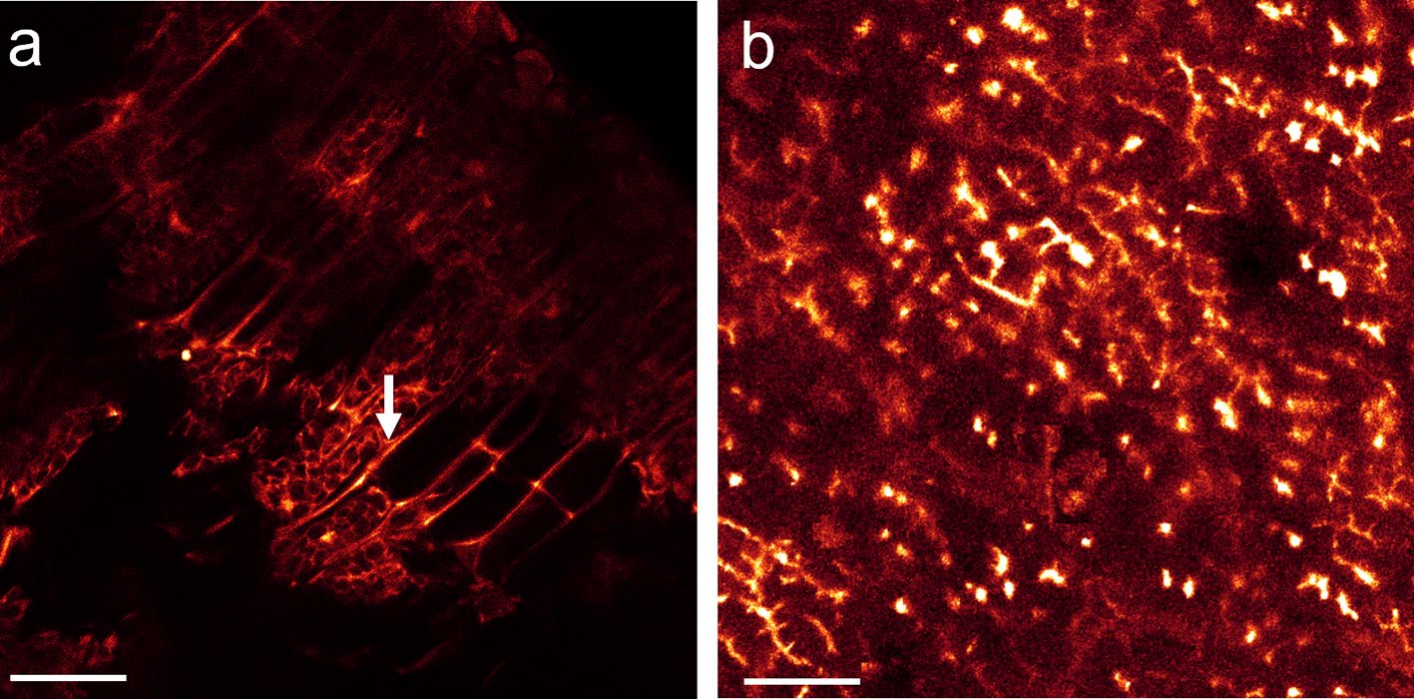
(**a**) Single image extracted from an xyz-stack, for fluorescently labeled ß-mannanase on SBM after 2 h incubation at room temperature. ß-mannanase predominantly adsorbed to intracellular domains enclosing the PSVs and to cell walls (marked exemplarily by white arrow). (**b**) Cell wall-like structures of the hull became fluorescent as well (z-projection of xyz-stack using the ImageJ plugin Local Z Projector v1.5.4). Scale bar 40 µm. See also video 6 in SI.

By using fluorescently labeled ß-mannanase we corroborated that ß-mannanase strongly adsorbed to the intracellular matrix enclosing PSVs and to the cell walls (Fig. 7a). Furthermore, it exhibited a high affinity for fragments of the soybean hull, in particular, for the cell wall structure (Fig. 7b).

**Figure 8:**
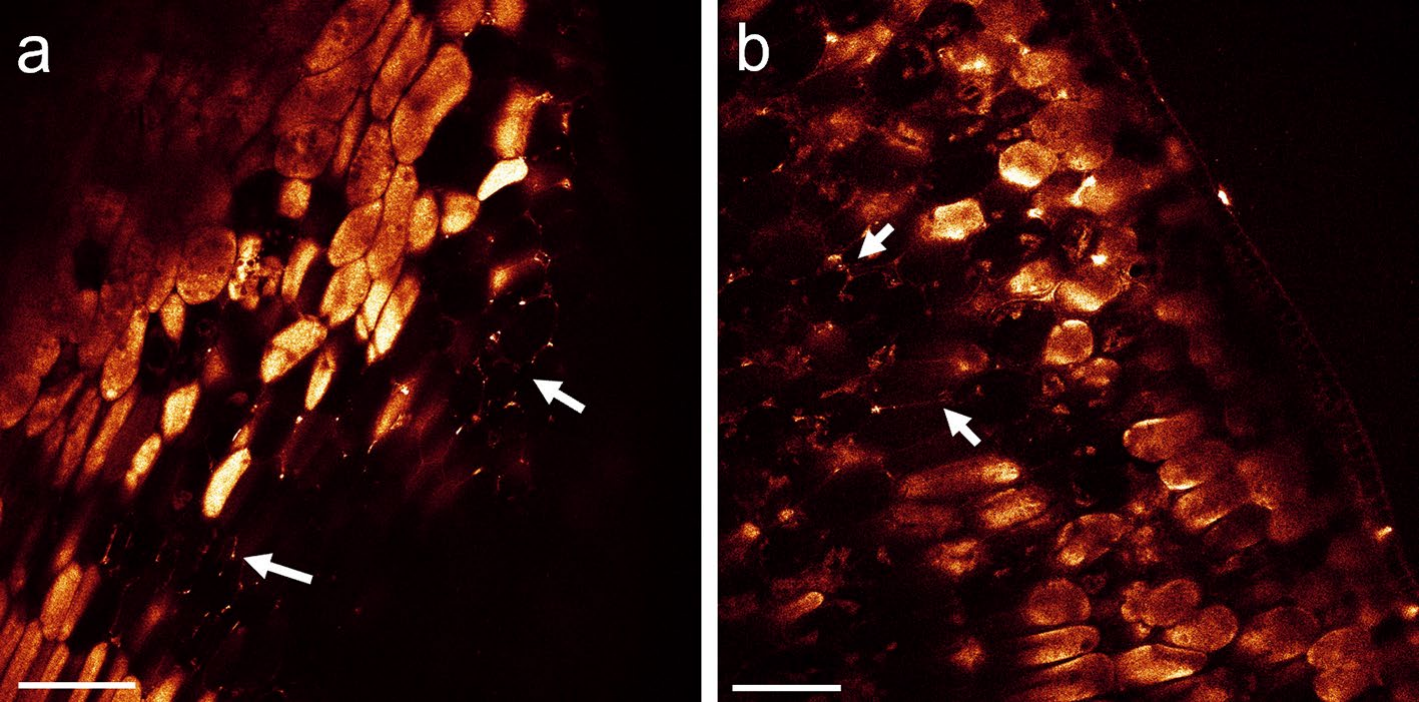
Single images of a xyz-stack for sections of soybean seeds in contact with fluorescently labeled ß-mannanase for 2 h after (**a**) chemical reduction and (**b**) without reduction. In both cases ß-mannanase adsorbed to the cytoplasm of cells almost uniformly and much weaker to the cell walls (marked by white arrow). The affinity of the labeled ß-mannanase for the sections was so strong that no fluorescent signal could be detected anymore outside of the section within the staining solution. Scale bar is 100 µm.

Furthermore, we verified that the chemical reduction process does not alter the adsorption behavior of fluorescently labeled ß-mannanase (Fig. 8a,b). For sections of soybean seeds, either chemically reduced or non-reduced, ß-mannanase exhibits a strong affinity for the sections as no fluorescence signal could be detected anymore in the aqueous solution around the section. The affinity for the cell wall structure was much lower than for the cytoplasm of the cells but independent of the chemical reduction step. In comparison to SBM the distribution of ß-mannanase was quite uniform in the cytoplasm.

As a further control experiment the effect of ß-mannanase, deactivated by heating it up for 30 min at 100 °C, was studied. The deactivated ß-mannanase did not show any fluorescence enhancement within the cell fragments of SBM and confirmed that only active ß-mannanase can lead to a fluorescence labeling of specific cell structures.

## Discussion

Typically, the mode of action of glycoside hydrolases is visualized either by using fluorescently labeled hydrolases or by studying hydrolase-triggered changes in structures, e.g. by visualization of changes in labeled or autofluorescent cellular structures. Here, a third concept was introduced which allows visualizing and tracking the activity of glycoside hydrolases directly by fluorescence labeling of their degradation products using aminooxy-functionalized fluorescent dyes. We demonstrated this concept by using ß-mannanase, acting on its target SBM substrate. Without the first step of this concept, i.e., the chemical reduction step prior to ß-mannanase treatment, mannanase-triggered changes in fluorescence labeling can hardly be detected. This is due to the fact that they are masked by the strong fluorescence background of native polysaccharides labeled at their reducing ends. Only if high concentrations of ß-mannanase are employed, weak fluorescence changes can be observed in-situ at the same location over time (not shown here) but because of potential spatial sample drifts over the course of the experiment, a clear identification of a ß-mannanase-triggered fluorescence increase at cellular structures is cumbersome. Therefore, to visualize the hydrolase activity in a more sensitive and reliable manner, it is necessary prior to the ß-mannanase treatment to convert the reducing ends of nearly all polysaccharide chains into a functional group, which is not able to react with an aminooxy functionality. This can be accomplished either by the reaction of reducing ends with a non-fluorescent aminooxy-bearing molecule (e.g. by aminooxy ethanol, not pursued here), or by a chemical reduction of the reducing ends to hydroxyl groups, which are not able to react with aminooxy groups^32,33^. The latter approach using a “mild” chemical reduction based on sodium borohydride was used as the first step in our concept and is frequently applied in life science in general^34^, e.g., to reduce the fluorescence background of tissue^35^ or to differentiate between exo-acting and endo-acting cellulases^36,37^. Sodium borohydride is supposed to modify only the reducing ends of the existing polysaccharides and generally does not reduce carboxylic acids, esters and amides in an aqueous medium without the assistance of further additives. Therefore, the reduction step is not supposed to affect other biomolecules, such as lipids and proteins^38^. Furthermore, chemical reduction of seeds and their meals is not supposed to modify their degradation behavior by endo-acting glycoside hydrolases as only the reducing ends of the polysaccharides are altered by this treatment. In fact, in the present study, no significant differences could be identified in the affinity of fluorescently labeled ß-mannanase to reduced or non-reduced sections of soybean seeds.

Apart from the reducing ends of polysaccharides, nucleic acids bear carbonyl groups and further carbonyl-bearing biomolecules can form upon oxidative stress by reactive oxygen species^39,40^ but chemical reduction of both the nucleic acids and oxidative stress-driven carbonyls impeded also the reaction with the aminooxy-bearing dye in the our study. Therefore, these components did not become fluorescent in our control experiments (without ß-mannanase) after the chemical reduction step and, thus, did not form a fluorescent cellular background.

In all experiments with ß-mannanase, the fluorescence signal rose at specific cellular structures, but no significant structural changes on the micrometer scale due to ß-mannanase-triggered hydrolysis became visible at the same time which hints to the superior sensitivity of this concept in relation to visualizing the structural degradation effects. Without ß-mannanase or by using deactivated ß-mannanase no fluorescence increase was detected. This directly verified that the fluorescence increase was caused by the action of ß-mannanase, at sites where ß-mannanase hydrolyzed ß-mannan-bearing polysaccharides and, thereby, formed new reducing ends. However, if the pH of the ß-mannanase solution was lowered to ~ 5 to mimic closer the gastric digestion in monogastrics, acid hydrolysis of various other types of polysaccharides (not only at ß-mannan-bearing polysaccharides) occurred in addition. This led to a strong and fast fluorescence enhancement at various cell structures, even in the control experiments without ß-mannanase but at pH 5 (not shown). Since our ß-mannanase shows the highest performance at pH 4, we could not visualize the ß-mannanase-triggered degradation at its optimum pH and can therefore assume that its performance is much stronger closer to the optimum pH. The combination of acid hydrolysis and ß-mannanase-triggered hydrolysis at lower pH can still be studied by this concept but the contribution of ß-mannanase alone cannot be evaluated easily.

The detection sensitivity for ß-mannanase-hydrolyzed polysaccharides is mainly limited by the background fluorescence of unbound aminooxy-bearing dye which lowers the imaging contrast but is not due to the fast oxime formation (reaction product of an aldehyde and an aminooxy group) catalyzed by aniline, which occurs in less than a minute and is therefore not rate-limiting in our case. By using non-fluorescent dyes, which turn on in fluorescence after reaction with the reducing end^41,42^, the sensitivity of the new visualization concept could be improved further as the background fluorescence would then be much weaker. Further limitations of our current visualization concept concern water-soluble fragments of ß-mannanase-hydrolyzed polysaccharides, which could not be detected as the labeled reducing ends were buried by the background fluorescence of the aqueous phase or were washed off later during the rinsing step in case of the ex-situ studies on soy bean seeds. However, a lower limit of the amount of released sugars could be estimated by quantifying the fluorescence intensity of the stained structures as at least one mannose unit was released by ß-mannanase activity before the remaining polysaccharide chain became fluorescently-labeled. Furthermore, the release of the remaining, labeled polysaccharide fragments could indicate a lower apparent hydrolysis rate of the ß-mannanase at the cellular substrate and, therefore, the real hydrolysis rate could be higher than was observed. However, we did not notice a decline in the fluorescence signal over time and, thus, we assume that the release of labeled fragments is at least lower than the rate of ß-mannanase-triggered hydrolysis.

The assessment of the strength of ß-mannanase activity on different cellular structures, like the intracellular matrix and the cell wall, depends on two factors. One is the staining efficiency by the aminooxy-bearing dye which is roughly on a similar level for cell walls and intracellular matrix (see video 3, SI). The second factor concerns the rate of hydrolysis of the polysaccharide chain by ß-mannanase, which can be limited by the accessibility of ß-mannanase. Therefore, one can interpret spatial differences of the fluorescence intensity to be roughly proportional to the local rate of hydrolysis by ß-mannanase.

Surprisingly, the novel visualization concept revealed that ß-mannanase was predominantly active at an intracellular matrix enclosing the PSVs and weaker at the cell walls for both soybean seeds and SBM, which is in contrast to the studies of *Ravn *et al. They reported only effects of their ß-mannanase on the cell wall using an immunofluorescence approach^15^, which could be either due to the application of a different type of ß-mannanase or, more likely, because their mannan-directed monoclonal antibody LM21 was not specific or not accessible to intracellular mannans.

For SBM the first activities were discovered intracellularly not earlier than 1 h and mainly after 24 h treatment when ß-mannanase was applied at low concentrations (80 ppm) at room temperature and at neutral pH. A noticeable activity could be observed at the cell wall, mainly at the middle lamella at the corner of adjoining cells. At the rest of the cell wall no activity could be discerned. Typically, pectins are the most abundant polysaccharides in the middle lamella of cell walls but our results hint to mannose-associated polysaccharides being part of it as well^43^. Only at much higher concentrations of ß-mannanase, primary cell walls were hydrolyzed additionally, likely representing other cell types of the soybean tissue than the ones where the middle lamella was attacked by ß-mannanase as we have seen no cells where both activities occurred at the same time. Apart from the endosperm cells, milled fractions of the hull were strongly affected by ß-mannanase as well.

By using fluorescently labeled ß-mannanase, only sites where it adsorbs to can be revealed but not its activity as we could not identify any structural changes on the cellular level. We found a close agreement of these adsorption sites with the sites where ß-mannanase is active, i.e. fluorescently-labeled ß-mannanase adsorbed onto the primary cell walls of endosperm cells, intracellularly at the matrix enclosing the PSVs and at the soybean hulls. The major difference in terms of adsorption sites for soybean meal and soybean seeds was the more uniform distribution intracellularly for soybean seeds. The PSVs in seeds can be seen only weakly in dark because spherical aberration reduces the axial resolution with depth as the distance of seed halves to the glass slide was much larger than for the milled SBM particles.

How can our findings on the ß-mannanase activity be interpreted? Endo-ß-mannanase is able to hydrolyze the backbone of pure mannans, galactomannans, glucomannans and galactoglucomannans^44^. The water-insoluble fraction of galactomannan is the prevalent group of hemicellulose, found, in particular, in the endosperm of the Leguminosae family, to which soybean belongs to. It is typically stored outside of the plasmalemma, both in the primary and secondary cell walls, providing a structural as well as an energy storage function^45^. The activity of ß-mannanase at the hemicellulose of the primary cell wall and at the hull of the soybean seed was reported in previous studies^12,15,46^ and is corroborated by our study with a stronger activity at the hull where galactomannans are more concentrated than in other fractions of soybean seeds^47^. It is speculated that pure mannans partially substitute cellulose in the cell wall and might instead provide the hardness and strength to the cell wall structure in soybean endosperm^9,48,49^. Beyond its enrichment in the cell wall *Herburger *et al*.* compiled strong evidence of further functions of mannans and coined the term of mannans as being the prime example of “moonlighting” polysaccharides^50^. For instance, in succulent plants acetylated mannans accumulate in intracellular compartments, likely providing energy or retaining water during periods of drought^51^. It is believed that galactomannans and other storage polysaccharides are secreted by the Golgi apparatus^52^ but evidence exists that galactomannans are synthesized as well in the intracisternal space of the rough endoplasmic reticulum (ER) in Faboideae, a subfamily of the Leguminosae^53^. In some Faboideae species, e.g. in fenugreek, the cytoplasm of endosperm cells can disappear entirely and the intracellular space becomes completely filled by galactomannans^9,53,54^. Moreover, in several other Faboideae species, gactomannans are potentially released directly to the cytosol, not via the Golgi apparatus, and accumulate inside the cell, potentially acting as an energy reservoir^50^. All these observations in related plant species corroborate our discovery of intracellular mannans in soybean seeds. Furthermore, evidence exists that polysaccharides in general are present as an intracellular matrix in soybeans: *Kasai *et al*.* and *Suryanarayana* found intracellularly a dense staining of endosperm cells in soybean using a Periodic acid–Schiff stain which is a typical staining method to detect polysaccharides^55,56^. Furthermore, lipid-linked, mannose-bearing oligosaccharides could act as structural lipids in soybean and potentially serve as intermediates in the synthesis of cell wall storage polysaccharides^57^. These lipid-linked oligosaccharides, bearing mannose units, are also involved as intermediates in the synthesis of β-conglycinin, one of the major storage proteins in soybean seeds, bearing a N-linked glycosylation which partially consists of alpha-linked mannose^58^. Therefore, we can interpret the surprisingly strong intracellular activity of ß-mannanase on galactomannans which are either not yet secreted to the cell wall or accumulate within the cytoplasm (ER and Golgi apparatus) and act as an additional energy reservoir. Indeed, ER and stacks of the Golgi apparatus are very abundant in plant cells and can form an extended network intracellularly which resembles the activity pattern of the ß-mannanase studied here^59,60^. Therefore, one can hypothesize that the utilization of a ß-mannanase is not only relevant in diets with low quality SBM containing a significant amount of soyhulls but also has a positive effect on higher quality SBM because it is active intracellularly within the endosperm, too.

## Conclusion

We demonstrated and validated our visualization concept to spatially resolve the activity for ß-mannanase. However, it can be readily applied to various others glycoside hydrolases and other types of substrates. Furthermore, it is conceivable to transfer this concept even to lipases and other classes of enzymes but then with a different fluorescence labeling approach. Using this method could reveal many previously unknown sites of enzyme activity and furthermore could be used to visualize the hydrolysable fraction of the enzyme-accessible structures in single cells and tissue. In combination with super-resolution fluorescence microscopy the spatial activity pattern of enzymes could be even visualized on the nanometer-scale and thus can provide more understanding of their mode of action.

By using this novel visualization concept, we can confirm that ß-mannanase is active at the cell wall and at the hull of soybean meal but, surprisingly, acts also strongly intracellularly where it potentially hydrolyzes intermediates for the synthesis of ß-mannans. Our results represent another piece of the puzzle for understanding the mode of actions of ß-mannanase. We found that the supplementation of ß-mannanase to soybean-based diets leads to the hydrolyzation of the enclosing matrix around the protein storage vacuoles and of cell walls. Thereby, the storage proteins could become more accessible for degradation by endogenous enzymes, which can lead to a better feed utilization in monogastric animals.

### Supplementary Information

Supplementary Information 1.

Supplementary Video 1.

Supplementary Video 2.

Supplementary Video 3.

Supplementary Video 4.

Supplementary Video 5.

Supplementary Video 6.

## Data Availability

All data generated or analysed during this study are included in this published article and its supplementary information files.
