# Supplementary material for: Spatial activity mapping of ß-mannanase on soybean seeds
Source: Sci Rep. 2024 Jan 10;14:1037. doi: 10.1038/s41598-024-51494-w (PMC10781726; doi:10.1038/s41598-024-51494-w)
Supplement: Supplementary file 1 — Supplementary Information 1. [file 41598_2024_51494_MOESM1_ESM.docx]

**Spatial activity mapping of ß-mannanase on soybean seeds**

**Markus Rueckel, Sven Janson, Arne Solbak and Anna Fickler**

**Supplementary info**

Video 1: xyz-stack of soybean half after 180 min treatment with 1000 ppm ß-mannanase in presence of 10 ppm of CF® 633 Dye Aminooxy, catalyzed by 1000 ppm aniline. The soybean half was not chemically reduced prior to the experiment. The first cell layer did not take up the fluorescent dye and therefore appears non-fluorescent. Field of view is 100 µm x 100 µm and stack depth is 66 µm.

Video 2: xyz-scan of a chemically reduced cotton fabric (by 0.4% sodium borohydride in water for 24h) after incubation with 100 ppm CF 633 Dye Aminooxy, 1000 ppm aniline and 100 ppm cellulase (from Aspergillus sp., Sigma-Aldrich C2605) for 45 min. and subsequent rinsing by water. Fluorescently labeled cellulose fragments were partially released from the cotton fiber and contributed to the fluorescence background. The cotton fibers were strongly stained and displayed the spatial pattern of cellulase activity. Without cellulase but under the same imaging conditions no fluorescence signal could be detected at the cotton fibers (not shown).

Video 3: xyz-scan of soybean meal (not chemically reduced) after 1 min staining by 100 ppm of CF® 633 Dye Aminooxy and 1000 ppm aniline and after 3 times centrifugation to wash out unbound dye. Stack depth is 36 µm and field of view is 300 µm x 300 µm. Both the intracellular structures and the cell walls were stained on a similar level. After 10 min staining the fluorescence level was not significantly higher.

Video 4: xyt-scan of chemically reduced SBM with 80 ppm ß-mannanase in presence of 100 ppm of CF® 633 Dye Aminooxy and 1000 ppm aniline. Duration of video sequence is 2h and field of view is 620 µm x 620 µm. Different time points from this video sequence were extracted for Fig. 5a-c.

Video 5: xyz-scan of chemically reduced SBM with 80 ppm ß-mannanase in presence of 100 ppm of CF® 633 Dye Aminooxy and 1000 ppm aniline after 24h. Stack depth is 103 µm and field of view is 247 µm x 247 µm. A single section was extracted for Fig. 5f.

Video 6: xyz-scan of soybean meal after 2h incubation with 100 ppm of fluorescently labeled ß-mannanase. Stack depth is 85 µm and field of view is 250 µm x 250 µm. A single section was extracted for Fig. 7a.
